# FORMAL SERVICE USE BARRIERS AND ITS IMPLICATIONS ON PREPAREDNESS IN DEMENTIA FAMILY CAREGIVERS

**DOI:** 10.1093/geroni/igad104.1371

**Published:** 2023-12-21

**Authors:** Francesca Falzarano, Megan McCarthy

**Affiliations:** Weill Cornell Medicine, New York City, New York, United States; Weill Cornell Medicine, New York City, New York, United States

## Abstract

Dementia family caregivers are critical to sustaining the long-term services and supports system in the US, yet the identification-assessment-referral pipeline to connect caregivers to needed resources remains siloed and complex. The use of formal services can serve as a mechanism to foster preparedness, resiliency, and enhance positive aspects of the caregiving role and ameliorate against adverse care-related outcomes. Despite resounding sentiment among caregivers expressing their need for relevant services, formal service utilization remains strikingly low. A greater understanding is needed regarding the multidimensional factors, stemming from within and beyond the direct care environment, influencing perceived ability to carry out care responsibilities. Thus, the purpose of this study was to examine the individual, psychosocial, and contextual characteristics associated with preparedness and self-efficacy among 46 dementia family caregivers (Mage=55.9, SD=16.3) who participated in a mixed-methods study examining facilitators and impediments influencing service utilization. Overall, 65.9% of participants reported receiving no formal preparation for their care responsibilities. Results showed that age, spousal kinship, longer duration of care, financial stress, more difficulty accessing formal services, and more negative service experiences were associated with lower feelings of perceived preparedness and self-efficacy. Findings underscore the need for more holistic, person-centered assessment to equip caregivers with the needed, relevant resources to enhance their ability to provide effective care.

